# RNF5: inhibiting antiviral immunity and shaping virus life cycle

**DOI:** 10.3389/fimmu.2023.1324516

**Published:** 2024-01-05

**Authors:** Junyi Ge, Leiliang Zhang

**Affiliations:** ^1^ Department of Clinical Laboratory Medicine, The First Affiliated Hospital of Shandong First Medical University and Shandong Provincial Qianfoshan Hospital, Jinan, Shandong, China; ^2^ Department of Pathogen Biology, School of Clinical and Basic Medical Sciences, Shandong First Medical University and Shandong Academy of Medical Sciences, Jinan, Shandong, China; ^3^ Medical Science and Technology Innovation Center, Shandong First Medical University and Shandong Academy of Medical Sciences, Jinan, Shandong, China

**Keywords:** RNF5, STING, MAVS, IRF3, SARS-CoV-2

## Abstract

RNF5 is an E3 ubiquitin ligase involved in various physiological processes such as protein localization and cancer progression. Recent studies have shown that RNF5 significantly inhibits antiviral innate immunity by promoting the ubiquitination and degradation of STING and MAVS, which are essential adaptor proteins, as well as their downstream signal IRF3. The abundance of RNF5 is delicately regulated by both host factors and viruses. Host factors have been found to restrict RNF5-mediated ubiquitination, maintaining the stability of STING or MAVS through distinct mechanisms. Meanwhile, viruses have developed ingenious strategies to hijack RNF5 to ubiquitinate and degrade immune proteins. Moreover, recent studies have revealed the multifaceted roles of RNF5 in the life cycle of various viruses, including SARS-CoV-2 and KSHV. Based on these emerging discoveries, RNF5 represents a novel means of modulating antiviral immunity. In this review, we summarize the latest research on the roles of RNF5 in antiviral immunity and virus life cycle. This comprehensive understanding could offer valuable insights into exploring potential therapeutic applications focused on targeting RNF5 during viral infections.

## 1 Introduction

Innate immunity serves as the first line of defense against viral infections, relying on various cellular mechanisms to detect and eliminate invading pathogens. The recognition of viral nucleic acids, particularly DNA, triggers a cascade of signaling events leading to the production of type I interferon (IFN) and other proinflammatory cytokines. This response is crucial for initiating antiviral defenses and shaping subsequent adaptive immune responses. However, precise regulation of these signaling pathways is essential to prevent excessive inflammation or autoimmunity.

Cytosolic DNA sensing is a critical aspect of antiviral immune responses, and the cyclic GMP-AMP synthase (cGAS)-stimulator of interferon genes (STING) pathway has been extensively studied in this context (1, 2). STING, also known as MITA, MPYS and ERIS (3–5), consists of the transmembrane (TM) at the N-terminal, which anchors STING on the endoplasmic reticulum (ER), mitochondria membrane, and mitochondrial-associated membrane (6), and the C-terminal spherical domain (7). Upon binding to cytosolic DNA in a sequence-independent manner, cGAS synthesizes cyclic GMP-AMP (cGAMP) (2, 8), whose cyclic dinucleotides (CDNs) activate the adaptor STING on the ER. STING then traffics from the ER to the Golgi complex and eventually to perinuclear compartments, during which process RNF5 recruits downstream signaling molecules, such as TANK-binding kinase 1 (TBK1), leading to the activation of transcription factors including nuclear factor-κB (NF-κB) and interferon regulatory factor 3 (IRF3), and subsequent induction of type I IFN and proinflammatory cytokines. Therefore, the cGAS-STING pathway has shown a significant defense role against a variety of DNA viruses (9), retroviruses (10, 11), and bacteria (12, 13). Besides DNA from these sources, the cGAS-STING is also activated by extracellular, mitochondrial, and nuclear DNA, including increased cytosolic DNA levels in cancers. With increasing studies providing mechanistic insights into the cGAS-STING pathway, it has been found that inappropriate activation of STING has been involved in the development of several diseases, including pulmonary fibrosis (14), senescence (15), neurodegeneration (16), cancer (16), amyotrophic lateral sclerosis (17). Moreover, research also explores the crosstalk between cGAS-STING and other innate immune pathways, which develops a balanced immune defense in the host.

In addition to STING, two crucial cytoplasmic pathogen recognition receptors, retinoic acid-inducible gene I (RIG-I) (18, 19) and melanoma differentiation-associated protein 5 (MDA5) (20) also play a role in recognizing viral RNAs. These receptors bind with viral RNAs and induce conformational changes and recruitment to the adapter protein mitochondrial antiviral signaling (MAVS, also known as Cardif, IPS-1, or VISA) (21–24). MAVS is a 540-amino-acid protein that contains a caspase activation and recruitment domain (CARD) that interacts with the N termini of RIG-I and MDA5, a proline-rich region (PRR), three TRAF-interacting motifs (TIMs), and a C-terminal transmembrane (TM) domain (21). MAVS triggers the formation of TRAF proteins (25), activates IRF3 and NF-κB, transmits the activation signal downstream, and induces the production of type I IFN and other antiviral molecules (21, 22).

Interestingly, studies have uncovered an intriguing interplay between RING finger protein 5 (RNF5, as known as RMA1) (26, 27) and both the STING and MAVS pathways (28, 29). As an E3 ubiquitin protein ligase in the ubiquitin modification system, RNF5 includes a classic RING domain which confers ligase activity and is anchored to the ER membrane through a single TM domain located within the C- terminal region. RNF5 has emerged as a key regulator in the modulation of these antiviral immune signaling pathways. Specifically, RNF5 has been shown to regulate the stability and function of both STING and MAVS (28, 29). RNF5 acts as a negative regulator of STING by promoting its ubiquitination and subsequent degradation (28). Through K48-linked polyubiquitination, RNF5 targets STING for proteasomal degradation, thereby limiting the duration and magnitude of STING-dependent immune responses. Similarly, RNF5 has also been implicated in the regulation of MAVS stability and function. It has been shown to promote the degradation of MAVS through ubiquitination, thereby dampening the downstream signaling events triggered by MAVS during viral infections (29). The dysregulation of RNF5-mediated regulation of STING and MAVS can have significant implications for host antiviral immunity. Aberrant activation or dysregulated expression of RNF5 may lead to compromised immune responses, allowing for increased viral replication and pathogenesis. Conversely, the regulation of RNF5 and its interaction with STING and MAVS presents potential therapeutic avenues for modulating immune responses and combating viral infections.

Given the fundamental role of STING and MAVS in triggering type I interferon signaling following infection, numerous viruses have developed ingenious strategies to counteract antiviral immunity through the modulation of RNF5. Pseudorabies virus (PRV), also known as Aujeszky’s disease virus or suid herpesvirus 1, an alpha-herpesvirus subfamily member, has evolved multiple evasion tactics to thwart host innate immune responses. Notably, the PRV envelope protein UL13, a novel viral immunoevasion protein, can collaborate with RNF5 to induce ubiquitination and degradation of STING, thereby inhibiting the antiviral immune response (30). Moreover, during infection with herpes simplex virus type 1 (HSV-1), RNF5 expression is upregulated in corneal tissues and corneal epithelial cells (31). This increase leads to a significant reduction in STING levels through ubiquitination and degradation. By promoting RNF5, the PB1 protein from the H7N9 virus facilitates K27-linked ubiquitination and recruits the selective autophagic neighbor of BRCA1 (NBR1) to aid in the degradation of ubiquitinated MAVS (32).

In this review, we aim to comprehensively explore the current understanding of the interplay between RNF5 and antiviral immunity, with a particular focus on its interaction with the cGAS-STING and MAVS pathways. We will discuss the regulatory mechanisms underlying RNF5-mediated ubiquitination and degradation of both STING and MAVS and highlight the implications of RNF5 dysregulation in viral pathogenesis. Additionally, we will delve into the strategies employed by viruses to exploit RNF5 for immune evasion. Finally, we will discuss potential therapeutic approaches targeting RNF5 to modulate immune responses and combat viral infections. Overall, the dynamic interplay between RNF5, STING, and MAVS holds great promise in advancing our knowledge of host-virus interactions and may provide opportunities for the development of novel antiviral strategies.

## 2 Immune antagonism of RNF5

The protein levels of STING, MAVS, IRF3 are delicately regulated through the ubiquitination mechanism to elicit antiviral immunity and avoid excessive harmful autoimmunity, with RNF5 acting as a negative regulator. Recent studies have shown that RNF5 promotes the degradation of STING, suggesting it as a potential target for treating pathological conditions such as cardiac hypertrophy (28). Additionally, RNF26 mediates a different type of polyubiquitination of STING, which is required for efficient antiviral response (33). However, many DNA viruses have evolved mechanisms to manipulate RNF5 to counteract the function of STING. Several host factors have been identified that regulate MAVS and immune responses by targeting RNF5. RNA viruses have also been shown to exploit RNF5 to degrade MAVS. IRF3, a transcription factor involved in antiviral responses, is another substrate of RNF5 (34). RNF5-mediated degradation of IRF3 serves to suppress innate immunity and promote the replication of some RNA viruses.

### 2.1 STING

**Figure 1:**
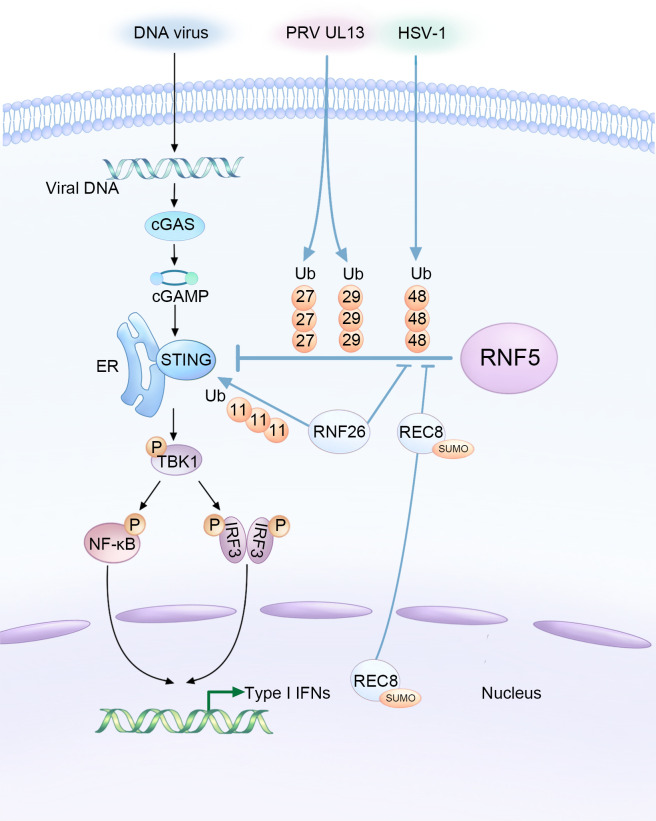
Complex interaction among DNA viruses, host factors, and RNF5 in the regulation of STING-mediated innate immunity during infections. HSV-1 and the UL13 protein of PRV facilitate the RNF5-mediated ubiquitination of STING, resulting in the inhibition of type I interferon production as a means to evade the innate antiviral response. Conversely, RNF26 and REC8 play a delicate role in suppressing the K48-linked ubiquitination of STING catalyzed by RNF5. This stabilization of STING protein enhances innate immunity, ultimately leading to the inhibition of DNA viral infections.

STING plays a critical role in the innate immune response against DNA viruses. Mice lacking STING have been shown to have impaired responses and decreased type I IFN production against various DNA viruses, such as HSV-1 and vaccinia virus (9). However, aberrant activation of STING can also lead to autoinflammatory and autoimmune diseases. Research has shown that the protein level of STING is regulated through the ubiquitination mechanism, with RNF5 acting as a negative regulator, which requires their respective TM domains (28). The intricate relationship between DNA viruses, host factors, and RNF5 in modulating STING-mediated innate immunity during viral infections is illustrated in **Figure 1**. Overexpression of RNF5 results in K48-linked ubiquitination of STING at K150, subsequently inducing a significant downregulation of STING. However, this RNF5-mediated degradation could be effectively reversed by the proteasome inhibitor MG132. Therefore, RNF5 targets STING for K48-linked ubiquitination and degradation through the proteasome pathway, exerting a negative regulatory effect on the antiviral response (28). Additionally, RNF5 has been found to suppress the development of pathological cardiac hypertrophy by potentiating the degradation of STING in cardiac myocytes, suggesting a potential treatment strategy for this condition (35).

Recent research has expanded our understanding of RNF5 regulation by demonstrating that in black carp, RNF5 promotes the degradation of black carp STING through the K48-linked ubiquitin-proteasome pathway. This provides new insights into the regulatory mechanisms involved in the appropriate immune response of black carp (36). Another E3 ubiquitin ligase, RNF26, mediates K11-linked polyubiquitination of STING as a reservoir of STING. Interestingly, RNF26-mediated polyubiquitination of STING also targets K150, which is targeted by RNF5 for K48-linked ubiquitination. Knockdown of RNF5 enhances RNF26-induced K11-linked polyubiquitination of STING, indicating a regulatory relationship between these two ubiquitin ligases. The balance between RNF5-mediated K48-linked ubiquitination and RNF26-mediated K11-linked polyubiquitination is crucial for efficient type I IFN and proinflammatory cytokine induction following infection (33).

Given the important role of STING in activating the type I IFN signaling pathway after infection, it is not surprising that many DNA viruses have evolved mechanisms to counteract STING’s function and facilitate their activities within host cells by manipulating RNF5. PRV employs various strategies to evade the host’s innate antiviral response. One of its tegument proteins, UL13, acts as a viral immune escape protein that partners with RNF5 to induce ubiquitination and degradation of STING, suppressing antiviral immunity (30). UL13 interacts with the cyclic dinucleotide (CDN) domain of STING and facilitates the binding of RNF5 to STING. Interestingly, RNF5 enhances UL13-related K27-/K29-linked ubiquitination of STING instead of K48-linked ubiquitination. The significance of K27-/K29-type linkage in this context is still largely unknown.

Furthermore, RNF5 expression was found to increase in corneal tissues and corneal epithelial cells during infection with HSV-1, which, like PRV, is a neurotropic herpesvirus. HSV-1 infection often results in corneal opacity and haze, with numerous neutrophils infiltrated the cornea, eventually leading to blindness due to inflammation and angiogenesis (37). RNF5 was shown to be constitutively expressed in corneal tissues and its expression increased upon HSV-1 infection, leading to a decrease in STING content through ubiquitination and degradation (31). Silencing RNF5 inhibited viral replication and reduced the number of inflammatory cells and the secretion of proinflammatory cytokines, thus alleviating corneal tissue injury. This suggests that targeting RNF5 could be a potential therapeutic approach for the treatment of HSV-1 infection.

### 2.2 MAVS

**Figure 2:**
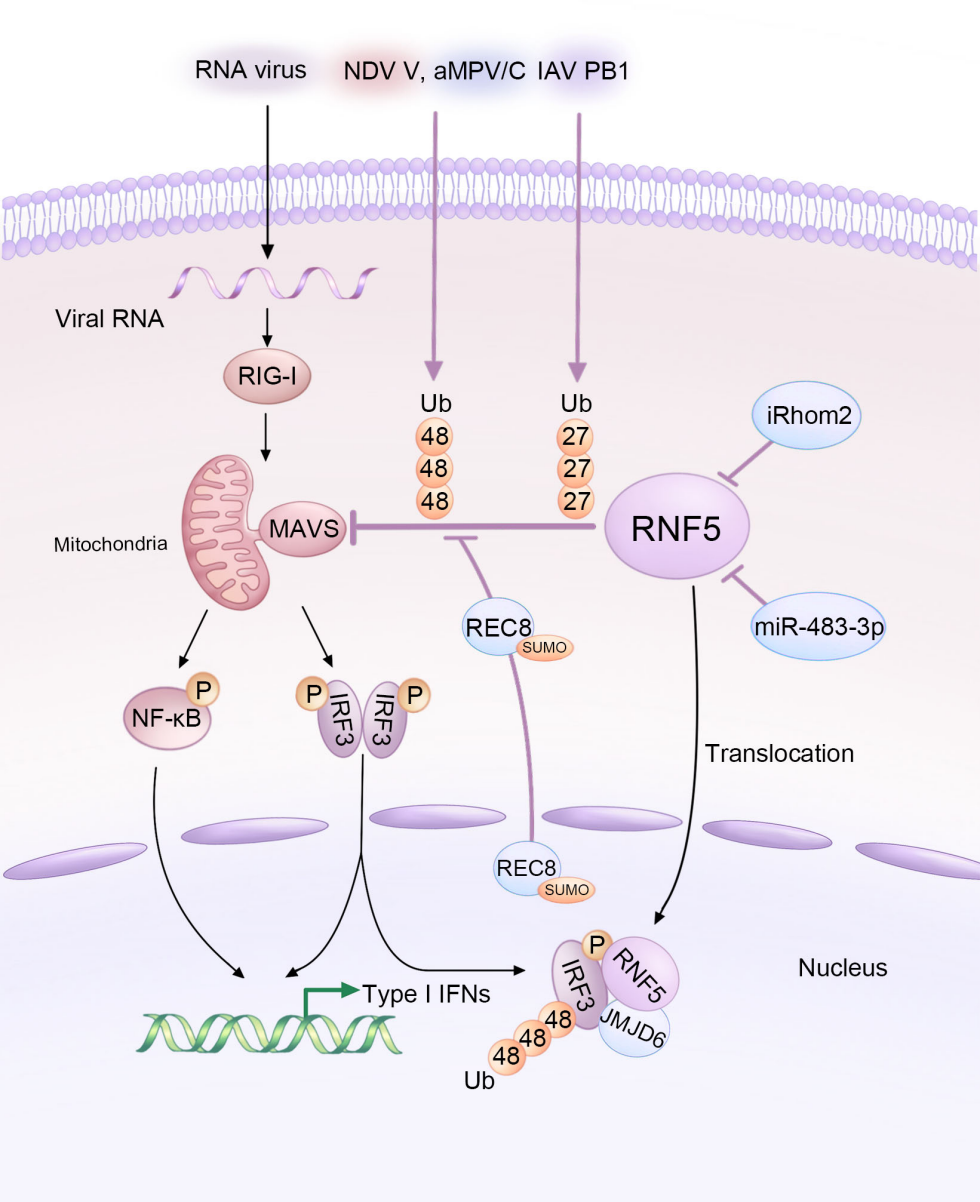
Diagram illustrating the modulation of MAVS-mediated signaling by RNA viruses and host factors through RNF5 during infections. The V protein of NDV, PB1 protein of IAV, and aMPV/C exploit RNF5 to orchestrate the ubiquitination of MAVS, thus inhibiting the type I interferon response. However, iRhom2 and miR-483-3p act as antagonists by decreasing the protein levels of RNF5. Additionally, REC8 downregulates the RNF5-induced ubiquitination of MAVS. Furthermore, JMJD6 functions as a negative immune regulator by degrading activated IRF3 in an RNF5-dependent manner.

The content and activity of MAVS are tightly regulated by RNF5 and other factors (38). The intricate interplay between RNA viruses, host factors, and RNF5 in modulating MAVS-mediated signaling during viral infections is summarized in **Figure 2**. Zhong et al. previously found the interaction between RNF5 and MAVS in a mammalian overexpression system (28) and further investigated the association later (29). The intermediate domain, along with either the RING or the TM of RNF5, and the C-terminal TM domain of MAVS are essential for the interaction. Coimmunoprecipitation experiments revealed a progressively strengthened association between the proteins at 6-12 hours post-infection, suggesting an early stage regulation that differs from that of RNF125 and PSMA7 (39, 40). In human HeLa cells, RNF5 catalyzes K48-linked ubiquitination of MAVS at K362 and K461, analogous to K363 and K462 in monkey Vero cells (41). Similar to STING, RNF5 targets MAVS for ubiquitin-mediated and proteasome-dependent degradation in the mitochondria, and it can also interact with MAVS in fish, indicating a novel negative regulatory factor of the RIG-I signaling pathway (42).

Several host factors have been reported to regulate MAVS and the immune response by targeting RNF5. ER-associated inactive rhomboid protein 2 (iRhom2) antagonizes the protein level of RNF5 in uninfected and early infected HEK 293 cells to maintain MAVS stability (43). iRhom2 mediates the self-association and auto-polyubiquitination of RNF5, impairing the ERAD of MAVS and promoting innate antiviral immunity (43). The function of iRhom2 might also be responsible for maintaining the stability of STING (44). In addition, lung-derived miR-483-3p, presenting at high levels in bronchoalveolar lavage fluid (BALF) exosomes, directly targets the 3’UTR region of RNF5, downregulating both its gene and protein levels, which enhances the RIG-I signaling pathway involving MAVS and further potentiates innate immunity to influenza virus infection (45). Rec8 meiotic recombination protein (REC8), a member of the structural maintenance of chromosome (SMC) protein partners, inhibits the degradation of both MAVS and STING by RNF5 after being SUMOylated at K30 and K530 and translocating from nucleus to the cytoplasm, stabilizing these two proteins during viral infection (**Figure 2**). The underlying mechanism still needs further investigation (46).

Like DNA viruses, various RNA viruses hijack RNF5 to ubiquitinate and degrade MAVS, aiding in their replication or evasion. For example, Newcastle disease virus (NDV), a member of the *Paramyxoviridae* family, significantly degrades MAVS at the post-translational level. The NDV V protein interacts with MAVS and targets its K48-linked ubiquitination and degradation through RNF5 (47). Avian metapneumovirus (aMPV), another member of the *Paramyxoviridae* family, also mediates MAVS K48-linked ubiquitination and degradation through RNF5. However, the mechanisms employed by aMPV are different from those of NDV (41). Influenza viruses (IAVs), single-stranded negative-sense RNA viruses, also exploit RNF5 to degrade MAVS. The PB1 protein of H7N9 serves as a negative regulator that destabilizes MAVS by promoting RNF5. PB1 enhances K27-linked ubiquitination and recruits the selective autophagy receptor NBR1 to facilitate the degradation of ubiquitinated MAVS via the selective autophagic pathway (32).

### 2.3 IRF3

As previously explained, both STING and MAVS activate TBK1, which in turn activates the transcription factor IRF3. IRF3 plays a crucial role as the downstream signal of MAVS. Upon viral infection, IRF3 undergoes phosphorylation-dependent dimerization, leading to the production of IFNs and other cytokines (48). Additionally, IRF3 acts as a direct effector in the transcriptional response, stimulating the synthesis of antiviral proteins (49). IRF3 has been identified as a substrate of RNF5 (34). This process involves the participation of Jumonji domain-containing protein 6 (JMJD6), which forms a tripartite complex with RNF5 and IRF3. JMJD6 plays a crucial role in recruiting RNF5 and IRF3, facilitating their translocation from the cytoplasm to the nucleus upon viral infection (34). Once activated, IRF3 is subsequently degraded by RNF5, which is recruited by JMJD6 (**Figure 2**). This mechanism serves to maintain the immune homeostasis and identify the role of JMJD6 as a negative regulator in the innate immunity during RNA viral infection, such as foot-and-mouth disease virus (FMDV) (34), which develops the previous notion that JMJD6 may be an unidentified third receptor of FMDV (50).

## 3 RNF5 in virus life cycle

The role for RNF5 in virus life cycle is gradually unfolding. Recent findings have revealed that RNF5 plays a role in limiting the replication and virulence of SARS-CoV-2 through the process of ubiquitination, particularly targeting the envelope (E) protein. Interestingly, in the study of Kaposi sarcoma-associated herpesvirus (KSHV) and its association with primary effusion lymphoma (PEL), a fascinating connection between RNF5 and Ephrin receptors has been discovered, opening up potential new avenues for the treatment of KSHV and management of PEL.

### 3.1 SARS-CoV-2

SARS-CoV-2, which belongs to the *Betacoronavirus* genus of the *Coronaviridae* family, has had an unprecedented and devastating impact on global society and the economy. The transmission of SARS-CoV-2 heavily relies on its viral proteins, including 4 structural proteins (spike [S], E, membrane [M], and nucleocapsid [N]), 16 nonstructural proteins, and 9 accessory proteins (open reading frame 3a [ORF3a], ORF3b, ORF6, ORF7a, ORF7b, ORF8, ORF9b, ORF9c, and ORF10) (51). While extensive research has been conducted on the binding of the S protein to the human angiotensin-converting enzyme 2 (ACE2) (52), the roles and regulatory mechanisms of the E and M proteins in the viral particle assembly process remain unclear.

**Figure 3:**
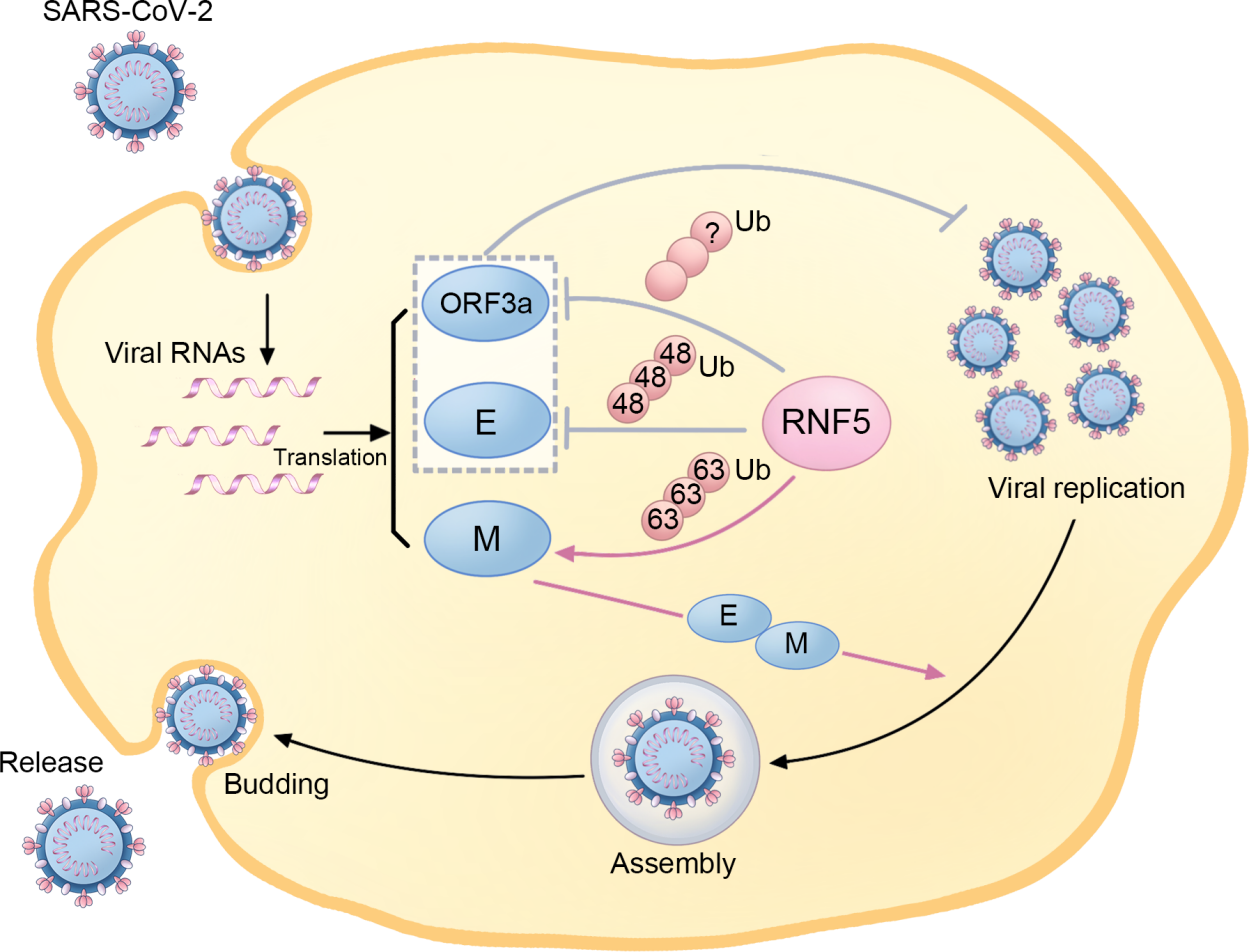
The regulatory role of RNF5 in the SARS-CoV-2 life cycle. During SARS-Cov-2 infection, RNF5 is responsible for K63-linked ubiquitination of the M protein, which enhances its interaction with the E protein and facilitates subsequent viral assembly, budding, and release. Furthermore, RNF5 also targets the ORF3a and E proteins for ubiquitination, thus suppressing SARS-CoV-2 replication and infection.

Ubiquitination and deubiquitination, two reversible modifications, have been implicated in the pathogenesis of SARS-CoV-2 (53, 54). It has been found that RNF5 is involved in the replication, assembly, budding, and release of SARS-CoV-2 (**Figure 3**). By mediating the K63-linked ubiquitination of M at the K15 site, RNF5 promotes the interaction of M’s NTD with E, stabilizing the M-E complex on the membrane, which mediates viral assembly and budding and ensures the uniform size of the viral particle. Additionally, this ubiquitin modification of M and the M-E interaction is necessary for the trafficking of M from the Golgi apparatus to autophagosomes, which facilitates virion release. Therefore, RNF5 is considered an emerging target for antiviral therapy as it facilitates virion release by mediating the ubiquitin modification of SARS-CoV-2 M (55).

However, some researchers have drawn adverse conclusions, suggesting a more complex role for RNF5 than previously thought. Recent evidence highlights that RNF5 mediates K48-linked ubiquitination of E at K63, leading to subsequent proteasomal degradation. This antagonistic action against SARS-CoV-2 replication and virulence limits the propagation of the virus. The RNF5 activator Analog-1 has been shown to significantly inhibit SARS-CoV-2 replication in infection models, making it a potential candidate for treating infections caused by related viruses. The levels of both RNF5 mRNA and protein are higher in adolescents than in older populations, and interestingly, the mRNA levels in mild SARS-CoV-2 patients exceed those in patients with severe symptoms, suggesting a potential role for RNF5 in reflecting the prognosis of SARS-CoV-2 patients (56). Due to the critical role of STING in chronic inflammation and functional decline during aging, the decrease of RNF5 in older populations, which leads to the declined degradation of STING, may also be involved in neurodegenerative processes in the elderly (16). Another important substrate of RNF5 in SARS-CoV-2 is ORF3a, which has been reported to inhibit autophagy activity and contribute to the infectivity of SARS-CoV-2 (57). RNF5 is responsible for the ubiquitination and degradation of ORF3a, thereby suppressing SARS-CoV-2 infection and replication (56).

The contradictory reports on the role of RNF5 during SARS-CoV-2 infection add complexity to the underlying mechanisms. These contradictions may be partly attributed to the diversity of cell types and the different rates studied, such as infection rate and virion release rate. It is possible that RNF5 may have diverse functions in different types of cells and viral stages. Additionally, the use of N or C-terminus-tagged RNF5 in experiments may also influence the results, as RNF5 is a tail-anchor TM protein with a C-terminal TM domain. Furthermore, some conclusions greatly rely on the utilization of specialized methods like virus-like particle (VLP) systems and super-resolution proximity labeling (SR-PL) rather than employing wild-type viruses, which might not completely reflect accurate status of viral infection and could potentially lead to different conclusions. In terms of the opposite influences of RNF5-mediated ubiquitination of M and E on SARS-CoV-2 infection, additional experiments have indicated that the activity of RNF5 against E is physiologically significant, while M has a higher affinity for RNF5 and appears to compete for RNF5, suppressing the degradation of E and promoting viral replication. It is important to note that the current understanding of the relationship between RNF5 and SARS-CoV-2 is limited, and further research is needed to elucidate the specific mechanisms and functional significance of this interaction. Considering the recognition of E from various SARS-CoV-2 strains, investigating the interplay between RNF5 and SARS-CoV-2 can provide valuable insights into the viral replication strategies and potential targets for board-spectrum therapeutic interventions.

### 3.2 KSHV

In addition to several crucial substrates involved in innate immunity, certain Ephrin receptors have been reported as novel substrates of RNF5. Ephrin receptors, consisting of 9 type-A members and 5 type-B members, have played diverse but contradictory roles in normal physiology and disease pathogenesis, particularly in tumorigenesis (58). Notably, specific Ephrin receptors have been identified to play significant roles in the entry and pathogenesis of KSHV (59, 60). KSHV is a critical factor in the development of PEL, a rare B-cell malignancy that mostly occurs in immunocompromised patients, such as individuals with AIDS (61).

**Figure 4:**
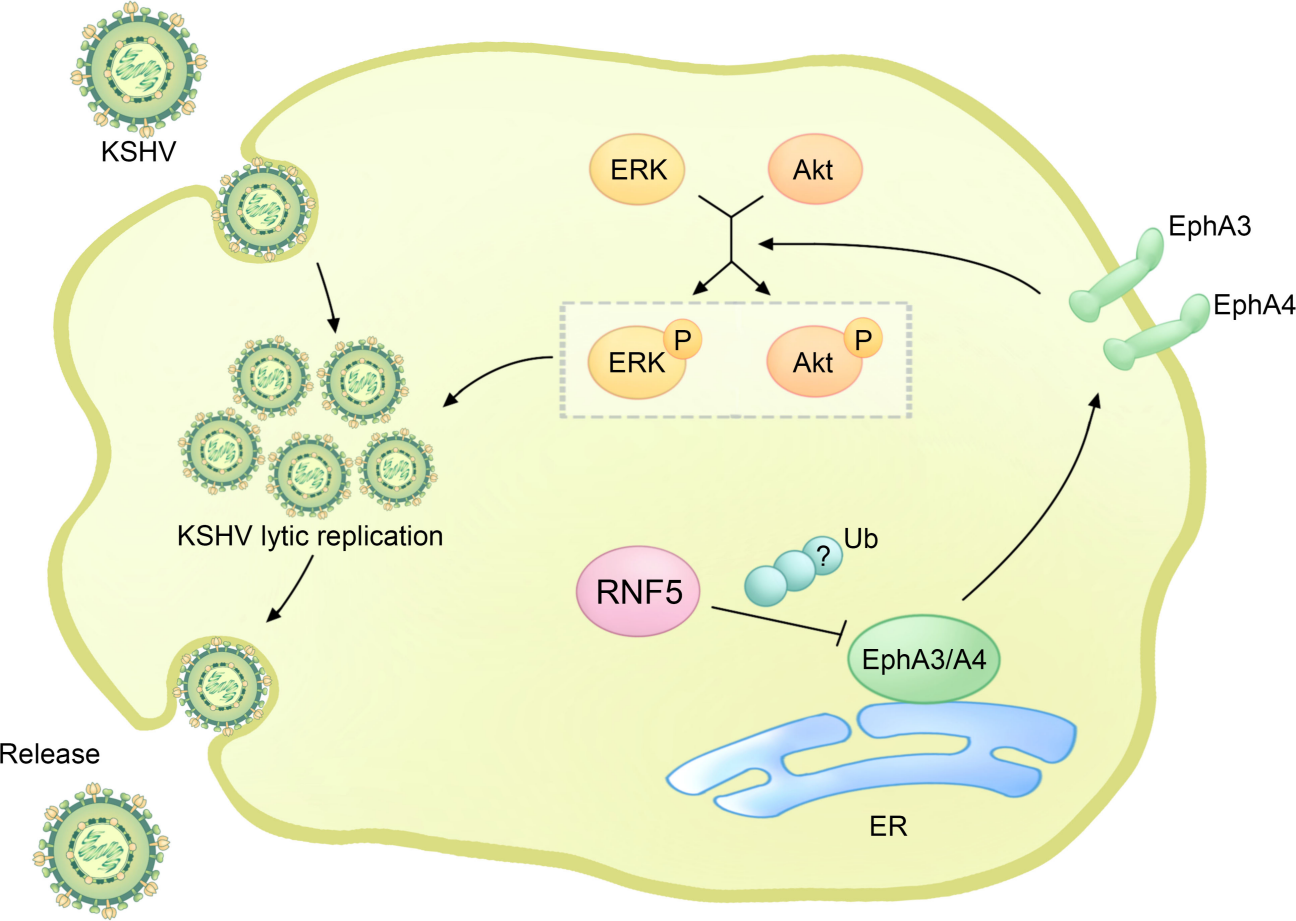
The regulatory role of RNF5 in the KSHV life cycle. During KSHV infection, RNF5 ubiquitinates and downregulates the levels of EphA3 and EphA4, further bolstering the activation of ERK and Akt, which could enhance KSHV lytic replication.

Interestingly, the phosphatidylinositide 3-kinase (PI3K)-Akt and extracellular regulated kinase (ERK)-MAPK pathways are constitutively activated by PEL, and the activation of ERK and Akt can enhance viral gene expression and viral loads in PEL cells, which is essential for KSHV replication (**Figure 4**) (60–62). RNF5 interacts with EphA3 and EphA4, promoting their ubiquitination and degradation, leading to the downregulation of EphA3 and EphA4 levels and subsequently upregulating ERK and Akt activation in PEL cells. Importantly, the introduction of inh-2, a specific RNF5 inhibitor, significantly suppresses KSHV lytic replication and the activation of ERK and Akt pathways by increasing EphA3 and EphA4 levels. Consequently, this downregulates the expression of multiple cellular pathways and KSHV viral genes in PEL cells (63). Collectively, these findings highlight the crucial role of RNF5 in both KSHV lytic replication and PEL tumorigenesis, suggesting the exciting possibility of utilizing RNF5 inhibition as a strategy for treating KSHV infection and PEL.

## 4 Conclusion and prospect

As an ER-associated E3 ubiquitin ligase, RNF5 demonstrates widespread expression in various cells and tissues, with the highest expression in breast cancer and melanoma (64). It induces selective ubiquitination of several proteins to regulate multiple physiological processes, indicating a pivotal regulatory role in ERAD (65), protein localization (66), autophagy (67), cancer procession (68, 69), and inflammation (70). Notably, RNF5 acts as an emerging negative regulator of antiviral innate immunity, which is the host’s first line of defense against virus infections. It functions in the K48-linked ubiquitination and degradation of STING and MAVS, essential adaptor proteins that resist DNA and RNA viral infections, respectively, as well as their downstream signal IRF3. Moreover, studies have also highlighted several host factors that collaborate with RNF5 to regulate innate immunity. For instance, RNF26, REC8, iRhom2, and miR-483-3p inhibit the degradation of STING or MAVS triggered by RNF5 via various mechanisms (33, 43–46). These factors maintain the stabilities of signaling proteins and promote the antiviral innate immune response. Conversely, JMJD6 contributes to IRF3 degradation in an RNF5-dependent way, achieving an opposite effect (34). In addition, viruses have developed ingenious strategies to hijack RNF5 for countering and escaping from antiviral defense. Certain DNA viruses, including neurotropic herpesviruses, PRV, and HSV-1, inhibit the antiviral effects of IFN I response by facilitating RNF5-mediated ubiquitination and degradation of STING/IRF3 signaling proteins. Similarly, RNA viruses such as IAV, NDV, and aMPV/C enhance MAVS degradation by usurping RNF5. In some cases, viral proteins play a pivotal role, including the UL13 protein of PRV, the V protein of NDV, and the PB1 protein of IAV, while mechanisms of other viruses still need further investigation. Surprisingly, UL13 and PB1 induce degradation of STING or MAVS through RNF5-mediated K27-linked ubiquitination, and PB1 promotes MAVS degradation via a selective autophagic pathway distinct from the canonical K48-linked polyubiquitination and subsequent proteasome pathway. These findings suggest that RNF5 can modulate different types of protein ubiquitination and degradation pathways in response to distinct viral infections. In general, RNF5 exhibits antagonistic roles in the cellular innate immune response to several viruses, with its abundance elaborately regulated by various host factors while simultaneously manipulated by RNA and DNA viruses.

There is growing evidence indicating that RNF5 is a promising drug target (63, 71), and the development of modulators and inhibitors of RNF5 holds significant importance. Notably, previous studies have shown that RNF5 suppression has no observable negative effects *in vitro* or *in vivo*, and mice lacking RNF5 expression show no altered phenotypes (67, 72). These findings highlight the potential for exploitation and druggability of RNF5 inhibitors. The first ever validated specific and selective inhibitor of RNF5, inh-2, belonging to the 1,2,4-thiadiazol-5-ylidene scaffold, was identified through ligand docking and virtual screening of the RING finger of the RNF5 structure. It also modulates ATG4B (67) and paxillin (66), which are both downstream targets of RNF5. However, the precise mechanism by which inh-2 exerts its activity through direct binding with RNF5 remains largely unknown (73). Further research has investigated the structure-activity relationships (SAR) of this class of compounds and identified compound 16 as a more potent RNF5 ligase activity inhibitor, directly binding to the RNF5 RING domain. These findings suggest that the 1,2,4-thiadiazolylidene scaffold may hold promise for the development of novel RNF5 inhibitors and drug-like derivatives (74). The original discovery of FX12, a small molecule that acts as both an inhibitor and degrader of the RNF5 ubiquitin ligase, provides a novel strategy for RNF5 inhibition. Rather than solely suppressing RNF5 E3 activity, FX12 directly binds to RNF5 and hijacks ERAD to initiate degradation of RNF5, possibly by altering RNF5’s normal structure to be recognized as a misfolded ER protein (75). However, while these studies have focused on the potential use of RNF5 inhibitors in the treatment of cystic fibrosis, limited research to date has explored their application in antiviral therapy (63). Therefore, further research into the effects of RNF5 inhibitors on antiviral immunity holds important implications.

Interestingly, RNF5 has recently been found to play a novel role in regulating SARS-CoV-2 viral replication and infection. However, there are contradictory reports on the subject. Initially, RNF5 was reported to mediate K63-linked ubiquitination of the M protein of SARS-CoV-2, thereby facilitating virion release, with autophagosomes possibly involved (55). In contrast, subsequent studies identified ORF3a and E proteins of SARS-Cov-2 as RNF5 novel substrates, revealing that RNF5 exerts a negative regulatory effect on viral replication and propagation, exerting anti-viral activities within host cells (57, 76). Furthermore, conflicting findings have been reported regarding the impact of SARS-CoV infection on RNF5 expression in different cell cultures. Given the contradictions in these studies, it is important to further investigate and fully comprehend the complex mechanisms of RNF5 in SARS-CoV-2, which could be facilitated through the application of RNF5 activators and inhibitors.

Exosomes are extracellular vesicles derived from cells that regulate cell-to-cell communication by transferring functional proteins and RNAs between cells (77). They contain functional proteins, mRNAs, and microRNAs (miRNAs). Interestingly, miR-483-3p is highly present in BALF exosomes in influenza virus-infected mice and directly targets the 3’ UTR of the RNF5 gene (78). The treatment of cells with miR-483-3p leads to the downregulation of RNF5 at both the gene and protein levels, enhancing the innate immune response against influenza. Considering RNF5’s role in mediating the degradation of both STING and MAVS, miR-483-3p may have broader implications beyond influenza, with great potential in limiting multiple respiratory viruses, including not only RNA viruses like parainfluenza virus and respiratory syncytial virus (RSV), but also DNA viruses like adenovirus. However, miR-483-3p can transfer between lung epithelial cells and vascular endothelial cells mediated through exosomes, promoting the expression of proinflammatory cytokine genes responsible for inflammation during influenza virus infection. Highly pathogenic avian influenza (HPAI) viruses have previously been reported to elicit dysregulation of proinflammatory cytokine production, resulting in multiple organ damage (79, 80). Therefore, further study is necessary to fully understand the mechanism behind exosomal miR-483-3p’s potential as an activator of the innate antiviral response.

From the above discussion, most existing studies have elucidated the multifaceted roles of RNF5 in innate antiviral immunity and virus life cycle. In addition, several host factors deliberately regulate RNF5 to maintain appropriate immunity, while viruses manipulate it for immune escape. These studies shed light on the broad-spectrum antiviral effects of targeting RNF5 on both DNA and RNA viruses, providing a theoretical basis for novel therapeutic strategies and the development of high-efficiency vaccines. However, due to the complex nature of its mechanism and interaction with proteins, more insights into RNF5 and the potential applications of RNF5 inhibitors in antiviral response will be instrumental in filling the gaps in our understanding of this field.

## Author contributions

JG: Writing – original draft. LZ: Conceptualization, Funding acquisition, Supervision, Writing – review & editing.
